# All-Printed Human Activity Monitoring and Energy Harvesting Device for Internet of Thing Applications

**DOI:** 10.3390/s19051197

**Published:** 2019-03-08

**Authors:** Shawkat Ali, Saleem Khan, Amine Bermak

**Affiliations:** 1 College of Science and Engineering, Hamad Bin Khalifa University, Qatar Foundation, Doha 5825, Qatar; sakhan3@qf.org.qa (S.K.); abermak@hbku.edu.qa (A.B.); 2 Electrical Engineering, National University of Computer and Emerging Sciences (FAST-NU), Islamabad 44000, Pakistan

**Keywords:** ZnSnO_3_, smart mat, Nano-generator, printed electronics, energy harvesting

## Abstract

A self-powered device for human activity monitoring and energy harvesting for Internet of Things (IoT) devices is proposed. The self-powered device utilizes flexible Nano-generators (NGs), flexible diodes and off-the-shelf capacitors. During footsteps the NGs generate an AC voltage then it is converted into DC using rectifiers and the DC power is stored in a capacitor for powering the IoT devices. Polydimethylsiloxane (PDMS) and zinc stannate (ZnSnO_3_) composite is utilized for the NG active layer, indium tin oxide (ITO) and aluminum (Al) are used as the bottom and top electrodes, respectively. Four diodes are fabricated on the bottom electrode of the NG and connected in bridge rectifier configuration. A generated voltage of 18 V_peak_ was achieved with a human footstep. The self-powered smart device also showed excellent robustness and stable energy scavenger from human footsteps. As an application we demonstrate human activity detection and energy harvesting for IoT devices.

## 1. Introduction

Electrical energy is needed everywhere in our life. from communication to food and travelling. To make human life more comfortable electronic gadgets, wireless connectivity and IoT devices have become part of life during the last few decades. In every IoT device electrical power is needed to operate the system but wired medium cannot always be established due to the large number of devices and their locations [1,2]. Most IoT devices are installed in places where a wired medium cannot be established as is the case of wearable sensors [3,4]. To solve this problem the electrical power should be generated within the application by energy harvesting from the ambient environment [5].

In recent years, energy-harvesting technologies that can scavenge various kinds of mechanical energy from the living environment have attracted increasing attention. Mechanical energy is among the most abundant and reliable energy sources in our daily life, which accompanies us regardless of the weather or temperature conditions like solar and thermoelectric energy [6]. Taking the forms of irregular air flow/vibration, ultrasonic waves, body movement, and hydraulic pressure, mechanical energy is ubiquitously available in our living environment. These mechanical energy sources have been converted into electrical energy by using piezoelectric/triboelectric cantilevers working in their resonating mode [7,8,9]. ZnO, BaTiO_3_, KNbO_3_, and sodium niobate (NaNbO_3_) have been reported for fabricating piezoelectric nanogenerators [10,11]. Sensors self-powered by nanogenerators are widely reported, for example, the use of triboelectricity for magnetic sensors [12], self-powered vehicle sensors [13], and self-powered temperature sensors [14]. These self-powered sensors are based on triboelectric nanogenerators and utilize materials which generate voltage based on the triboelectric effect. Piezoelectric materials are relatively more stable and do not require a large device size to operate as a voltage generator. Among the piezoelectric materials, zinc stannate (ZnSnO_3_) has been extensively studied because it is a piezoelectric and ferroelectric material with an electrical and structural ordering temperature well above room temperature (up to 700 °C) [15,16]. Furthermore, increased interest has developed around ZnSnO_3_ because of its symmetry-dependent properties such as piezoelectricity, ferro-electricity, pyro-electricity, and second-order nonlinear optical behavior that originate from its non-centrosymmetric properties [17]. In addition, its physical properties can be modulated by controlling the morphology, dimensions, crystallinity, curing temperature and preparation methods [18]. Zinc stannate is extensively used for NG applications and has shown promising performance in energy harvesting [19,20,21]. Utilizing NGs in combination with printed rectifiers and capacitors in order to store the produced power on the same system is crucial for IoT applications [22]. This will reduce need for extra connecting wires and losses at the same time that it reduces the physical size of the system.

**Figure 1:**
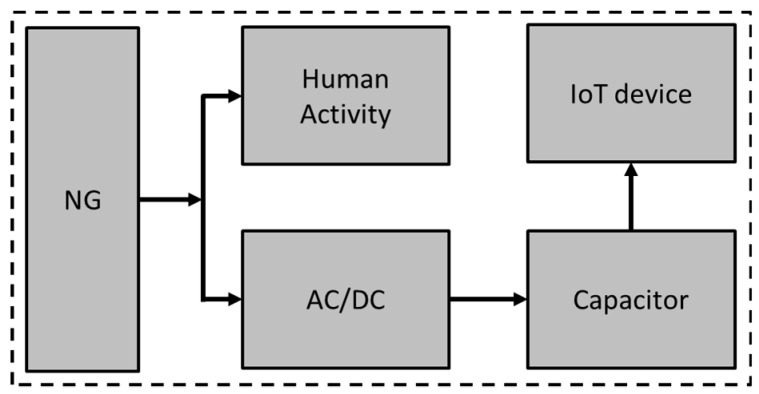
Block diagram of the people counting and motion sensor.

In this research work, a self-powered system that harvests energy from human footsteps and stores it on a capacitor available on the same system is proposed. A ZnSnO_3_ and PDMS blend is utilized for the fabrication of s NG that provides an 18 V peak voltage. The blended material is cast on ITO-coated PET and a top electrode of aluminum foil is placed on the NG active layer. Output is taken from the top and bottom electrodes through connecting wires. Printed diodes (Ag/ZnO/PEDOT:PSS/ITO) with high rectification ratio are fabricated on a patterned ITO-coated PET substrate and connected in bridge rectifier configuration. All components are connected in such way that the NG generates an AC voltage and the bridge rectifier converts the generated AC power into DC and the capacitor store this power for IoT applications. Other than the energy harvesting application, the AC spikes generated by the NG device provide useful information that can be used for human activity monitoring such as walk direction, running, walking and people counting applications. This work can be good basis for the self-powered systems that not only generate power from human activity but also provide information about that human activity. Figure 1 shows a block diagram of the self-powered human activity and energy harvesting system. The Nanogenerator generates voltage spikes and feeds them to a rectifier circuit where the AC voltage is converted to DC. At the same time, voltage spikes are fed to the counter circuit where spikes are analyzed in the time domain for human activity detection. The DC voltage from the rectifier circuit is then supplied to a capacitor for storage purposes. Voltage from the capacitor is supplied to the IoT device for its operation.

## 2. Materials and Methods

**Figure 2:**
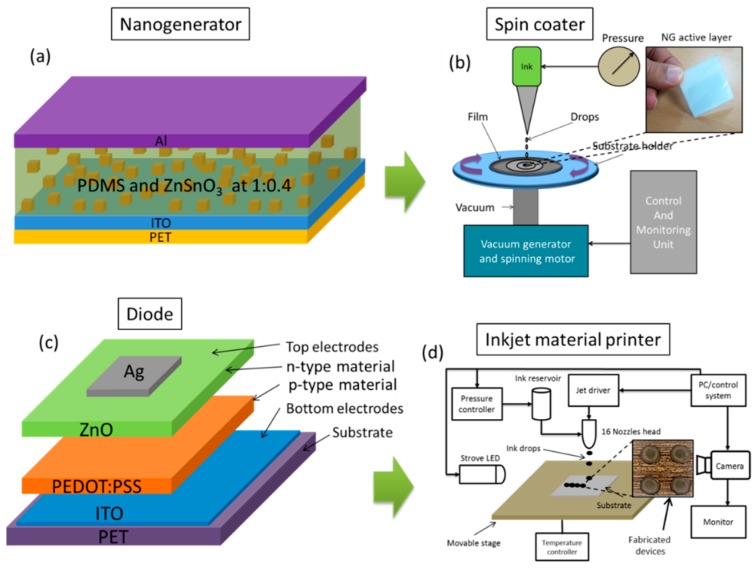
(**a**) NG layout diagram fabricated layer by layer. (**b**) NG fabrication with a spin coater. (**c**) Materials layout diagram of the diode device. (**d**) Schematic diagram of the inkjet material printer.

Zinc stannate powder ZnSnO_3_ (CAS 12036-37-1) was purchased from Go Yen Chemical (Kaohsiung, Taiwan) and PDMS Sylgard 184 was purchased from Dow Corning (Seoul, Korea). NG active layer material was prepared as follows: the two PDMS base and curing agent components were mixing in a 10:1 ratio and then ZnSnO_3_ powder (40% by wt.) was added to the PDMS solution and mixed for 10 min [16]. The mixed solution was placed in a vacuum chamber for 20 min for degassing. Figure 2a shows a diagram of the NG layout fabrication steps. It is a bottom to top fabrication process, where first the bottom electrode will be deposited and then the active layer followed by the top electrode. The active layer solution was deposited on ITO-coated PET through a spin coater as shown in the diagram in Figure 2b. During the spin casting of the solution, the machine operation parameters were a spinning speed of 500 rpm, spinning time 60 s with a 5 s ramp. After the deposition of the active layer, it was kept at 100 °C for 1 h. The top electrode of aluminum (Al) was placed by cutting aluminum foil according to the dimensions of the NG active layer. The optimum size of the NG was 3 × 3 cm^2^ in order of generate a high peak voltage. Copper wires were connected with the bottom and top electrodes of the NG by using silver epoxy.

**Figure 3:**
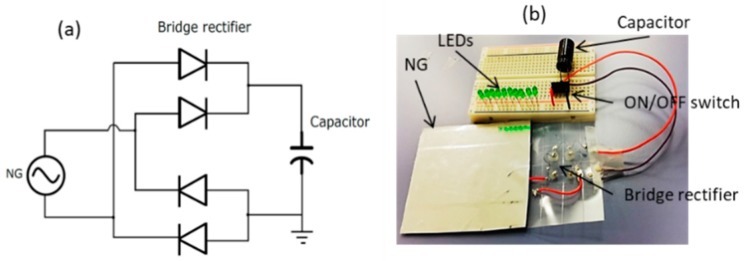
(**a**) Schematic diagram of the NG connected with bridge and storage capacitor. (**b**) Digital photograph of the fabricated system.

The diode device structure is ITO/PEDOT:PSS/ZnO/Ag as shown in Figure 2c. Four (4) diode devices were fabricated on the same substrate of the NG bottom electrode using a DMP 2850 inkjet material printer as shown in Figure 2d. p-type (PEDOT:PSS) and n-type (ZnO) materials were filled in two different cartridges (3 mL each). First, a PEDOT:PSS layer was deposited on ITO-coated PET and cured at 120 °C for 60 min. Then a ZnO layer was deposited over the cured PEDOT:PSS layer and cured at 120 °C for 60 min. Silver epoxy was used to attach the top electrode. After the fabrication of individual devices, they were connected in bridge configuration as shown in Figure 3a by using bonding wires and silver epoxy. Figure 3b shows a photograph of the system after making the interconnections. The bridge rectifier is fabricated on the bottom electrode of the NG and the capacitor is placed on a Vero board. Ten (10) LEDs are connected through an ON/OFF switch to the capacitor.

## 3. Results

**Figure 4:**
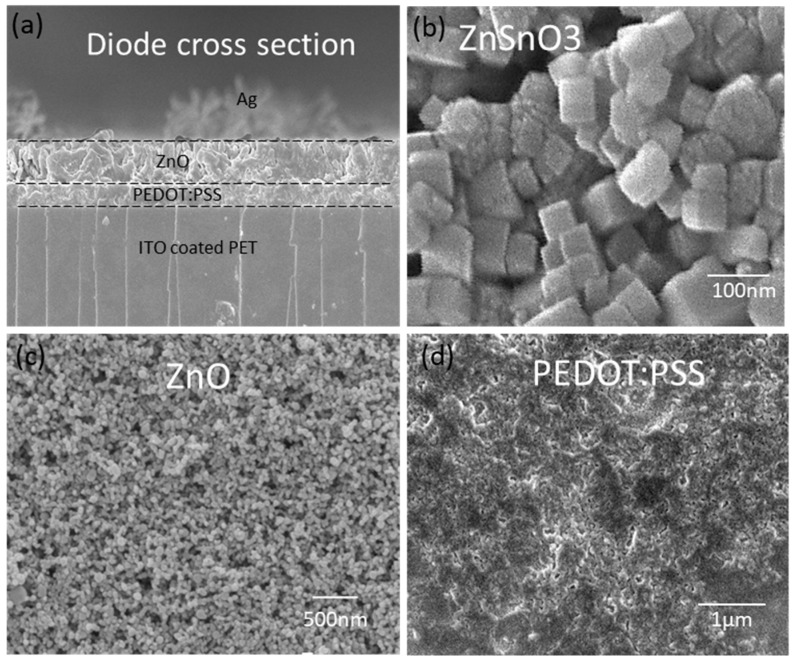
(**a**) Cross sectional view of the ITO/PEDOT:PSS/ZnO/Ag device. (**b**) Zinc stannate. (**c**) ZnO thin film SEM image. (**d**) PEDOT:PSS thin film SEM image.

Thin films of the devices were analyzed with scanning electron microscopy (SEM) in order to be sure of their mechanical characteristics. Figure 4a shows a SEM image of the diode device consisting of three layers deposited over the ITO-coated PET substrate. It can be seen that all layers are properly deposited and almost homogenous in thickness throughout the area. Figure 4b shows a SEM image of the ZnSnO_3_ nanocubes, where the cubic shape of the ZnSnO_3_ particles can be verified. The shape of the ZnSnO_3_ plays an important role in the piezoelectric behavior. If the shape is not cubic the efficiency of the NG will be low as the resultant charge produced on each cube will not located at the opposite ends of the particle but rather it will have a round shape. Figure 4c shows a SEM image of the ZnO, where ZnO nanoparticles can be seen. The ZnO film is deposited uniformly and is amorphous. Figure 4d shows a SEM image of the PEDOT:PSS material, which looks like a uniform film since it is a polymer material and hence no particles are present. It can be seen that the PEDOT:PSS film is deposited uniformly and cured at an appropriate temperature to avoid any cracks.

**Figure 5:**
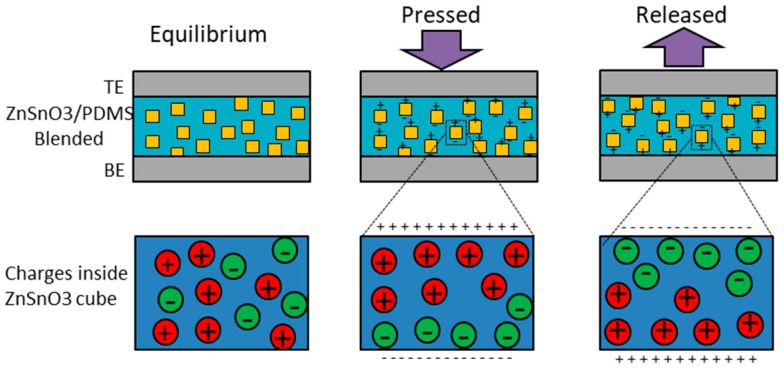
Working mechanism of the ZnSnO_3_:PDMS-based nanogenerator.

ZnSnO_3_ nanocubes are piezoelectric materials and generate voltage when a force is applied on them. ZnSnO_3_ cubes are embedded in the PDMS in order to provide them with stability in terms of deformability and elasticity, especially when it fabricated at such a low temperature, i.e., 120 °C. When processed at low temperature ZnSnO_3_ cannot be crystalline hence the PDMS provides support to the ZnSnO_3_ to make a film that is robust against pressing cycles. The blending weight ratio of ZnSnO_3_:PDMS is very important in determining the output voltage generation as a result of the force applied on it [16]. When the composite film is pressed, individual nanocubes of ZnSnO_3_ generate a voltage. As the nanocubes are spread over the entire film of PDMS, hence their generated charges are transferred to the electrodes through the PDMS. Figure 5 shows the working mechanism of the proposed nanogenerator. Under equilibrium conditions there is no net charge on the electrodes of the device and hence there is no electrical output from the device. When an external force is applied on individual nanocubes, positive and negative charges are produced at the opposite ends of the cube. These charges are added in series and appear at the electrodes through the PDMS medium. As a result of the accumulation of opposite charges at the electrodes a potential difference is experienced where the top electrode is positive and the bottom electrode is negative. When the force is released from the NG, the opposite phenomenon occurs and negative charges are accumulated on the top electrode and positive ones on the bottom electrode. The rate of change and amount of the applied force also play an important role in the generation of voltage. A single cube of ZnSnO_3_ is illustrated in the inset of Figure 5, where at equilibrium the charges inside the cube are randomly distributed and the net charge at the ends of cube is zero. When the cube is pressed positive and negative charges are aligned at the opposite ends of the cube. Similarly, when the force is released, the charge distribution is reversed at the ends of the cube.

Individual devices were characterized by using a B5100 semiconductor analyzer (Agilent, Santa Clara, CA, USA) in combination with a probe station. The ITO/PEDOT:PSS/ZnO/Al diode device was placed on the platen of the probe station and probes were connected to the anode and cathode. A −6 to +6 V sweep was applied and the current against the voltage sweep was recorded.

**Figure 6:**
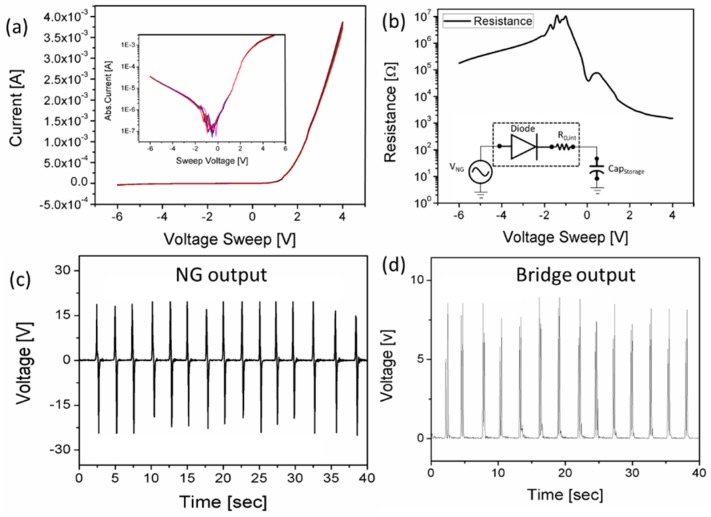
(**a**) I-V curve of the diode. The inset shows the absolute current graph of the diode. (**b**) Diode resistance against the sweep voltage. (**c**) NG output voltage against human footsteps applied for 40 s. (**d**) Output voltage at the bridge rectifier.

Figure 6a shows the current-voltage (I-V) analysis of the diode device, where it can be seen that the reverse current is almost eliminated from 0 to −6 V which shows the good rectification properties of the device. In the inset of Figure 6a the absolute I-V graph is shown, where the reverse current is shown on a semi-log scale, and the maximum value of the reverse current at −6 V is 2.5 × 10^−5^ A, whereas on the positive side at 4 V the current is 2 × 10^−3^ A. The difference between the reverse and forward peak current is 8 × 10^−3^ A. Figure 6b shows the resistance analysis of the diode device against the voltage sweep. The internal resistance of the diode is very important to know when using it for rectification purposes. If the internal resistance of a diode is high it will drop voltage proportionally to the current passing through it. This is the internal voltage drop of the diode and results in a lowering of the overall system’s efficiency. On the other hand, the internal resistance also plays an important role in low current applications, i.e. it limits the current drained from the NG and provided to a capacitor at the output, hence prevent any loading effect on the NG. In our case the resistance of the diode at −6 V is 2 × 10^5^ Ω and at 4 V the resistance decreases to 1.8 × 10^3^ Ω as shown in Figure 6b. This internal resistance plays an important role in limiting the current while charging the storage capacitor, and a schematic diagram is shown in the inset of Figure 6b. The NG was placed on the floor and wires were connected to semiconductor analyzer for recording the voltage generated by footsteps. The NG was measured for 40 s as shown in Figure 6c, the output voltage is 18 V on average against normal footstep tapping. Figure 6d shows the voltage at the output of the bridge rectifier where the negative peaks are also converted into positive peaks and represent a DC voltage. 

The self-powered device was characterized for its energy harvesting potential for IoT applications. The energy harvesting feature was tested on green LEDs. A 32 V, 100 μF capacitor was connected to the output of the bridge rectifier, 10 green LEDs were connected in parallel to the capacitor through an ON/OFF switch as shown in Figure 6a.

**Figure 7:**
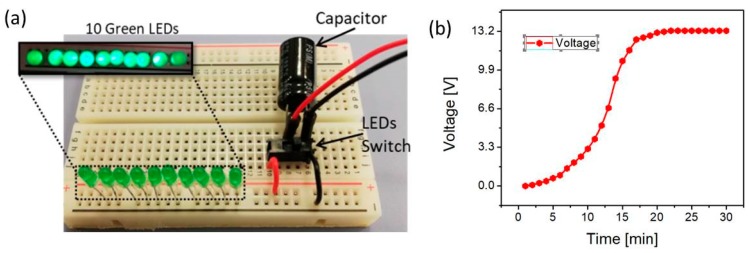
(**a**) 10 green LEDs glowed with harvested energy. (**b**) Harvested voltage across the capacitor.

The NG was tapped with footsteps for 30 min while the switch was OFF to prevent current flow through the LEDs and charge the capacitor. The 10 LEDs were connected in parallel to the output of the capacitor; it was observed that LEDs glowed for 1 sec as shown in Figure 7. Since IoT devices consume extremely low power and most of the time they stay in sleep mode, this harvested power is sufficient to power IoT devices. The capacitor charging voltage is shown in Figure 7b, where the maximum voltage stored on the capacitor after 30 min was 13.2 V. 

**Figure 8:**
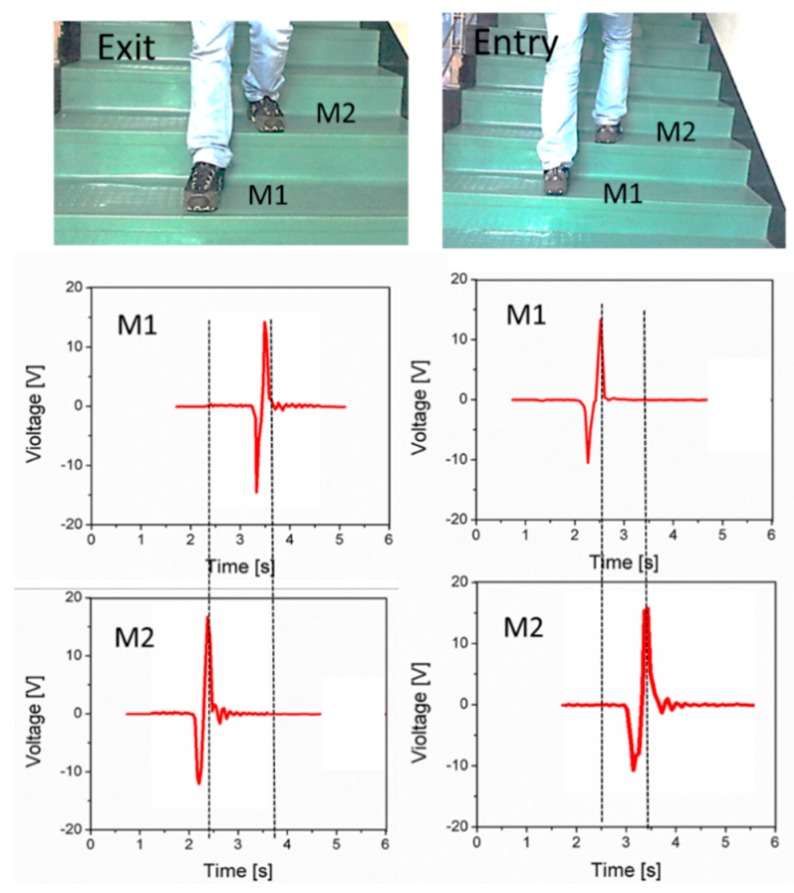
People counting application of the proposed device, in the Exit scenario: M2 is pressed prior to M1 the voltage spikes occurs at 2.5s for M2 and 3.5s for M1 in the time domain. In the Entry scenario: M1 generates voltage spike at 2.5s while M2 at 3.5s.

Another application of the smart device is human activity monitoring. For this test, two sensors were placed as M1 and M2 (M1 and M2 represent mat1 and mat2) at the entrance of a building on the stairs. Whenever M1 or M2 are tapped with footsteps they generate voltage spikes. In this scenario if M1 is pressed prior to M2, this will count as one person having entered the hall. Similarly, if M2 is pressed first and then M1, this will count as one person leaving the hall. The people counting scenario is shown in Figure 8, where during the “Exit” period the persons first step on M2 and then M1. In the result it can be seen M2 generates a voltage spike before M1. These two voltage spikes are compared in the time domain by using a microcontroller which shows this is an increment of one person in the hall. On the other hand, if a person steps on M2 first, it will generate a voltage spike prior to M1. These two spikes are compared in time domain by a microcontroller and represents the exit of one person from the hall.

**Figure 9:**
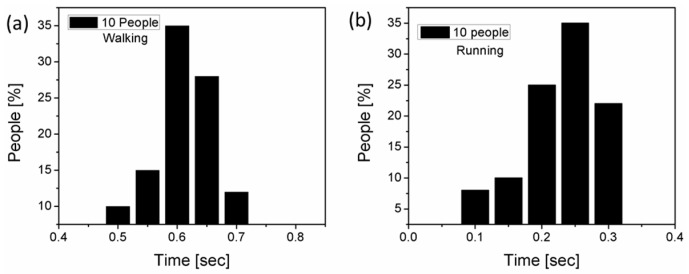
(**a**) “Walk” data of 10 people. (**b**) “Run” data of 10 people.

Human running and walking activity: Two sensors can be used for run/walk activity monitoring of humans. Under footsteps, each of the NGs generates voltage spikes; in general, both sensors were placed at a certain distance from each other so that a human can step on them easily. If a person is walking, the smart mats will generate voltage spikes with a certain time delay according to the rate of the footsteps. If the delay is less than the normal human being walking time period (1 to 2 steps per second) it will count as running. During normal walking, the step time for 10 people was 0.5 to 0.7 s/step and the running step time 0.1 to 0.3 s/step as shown in Figure 9. Data was collected from 10 different people with different walking/running styles. There is a prominent difference in the data between walking and running on the time scale, hence it is very easy to detect with a microcontroller instead of a computer-based signal processing system whether a person is walking or running.

## 4. Conclusions

In summary, a self-powered device for IoT energy harvesting and human activity monitoring applications is presented. The self-powered device consists of zinc stannate and PDMS-based nanogenerators, a printed bridge diode fabricated on an ITO-coated PET substrate and an off-the- shelf capacitor. A single NG produced 18 V by pressing with a footstep. The bridge diode converts the generated AC into DC power and stores it on a capacitor. AC spikes are further utilized to determine human activities such as running, walking, moving direction and people counting in combination with an Arduino microcontroller. The results are promising and can be good basis for self-powered sensors and energy harvesting devices for IoT applications.
